# Supplementary material for: Environmental Transmission of Symbionts in the Mangrove Crabs Aratus pisonii and Minuca rapax: Acquisition of the Bacterial Community through Larval Development to Juvenile Stage
Source: Microorganisms. 2024 Mar 25;12(4):652. doi: 10.3390/microorganisms12040652 (PMC11052079; doi:10.3390/microorganisms12040652)
Supplement: Supplementary file 1 [file microorganisms-12-00652-s001.zip › microorganisms-2364702-supplementary.pdf]

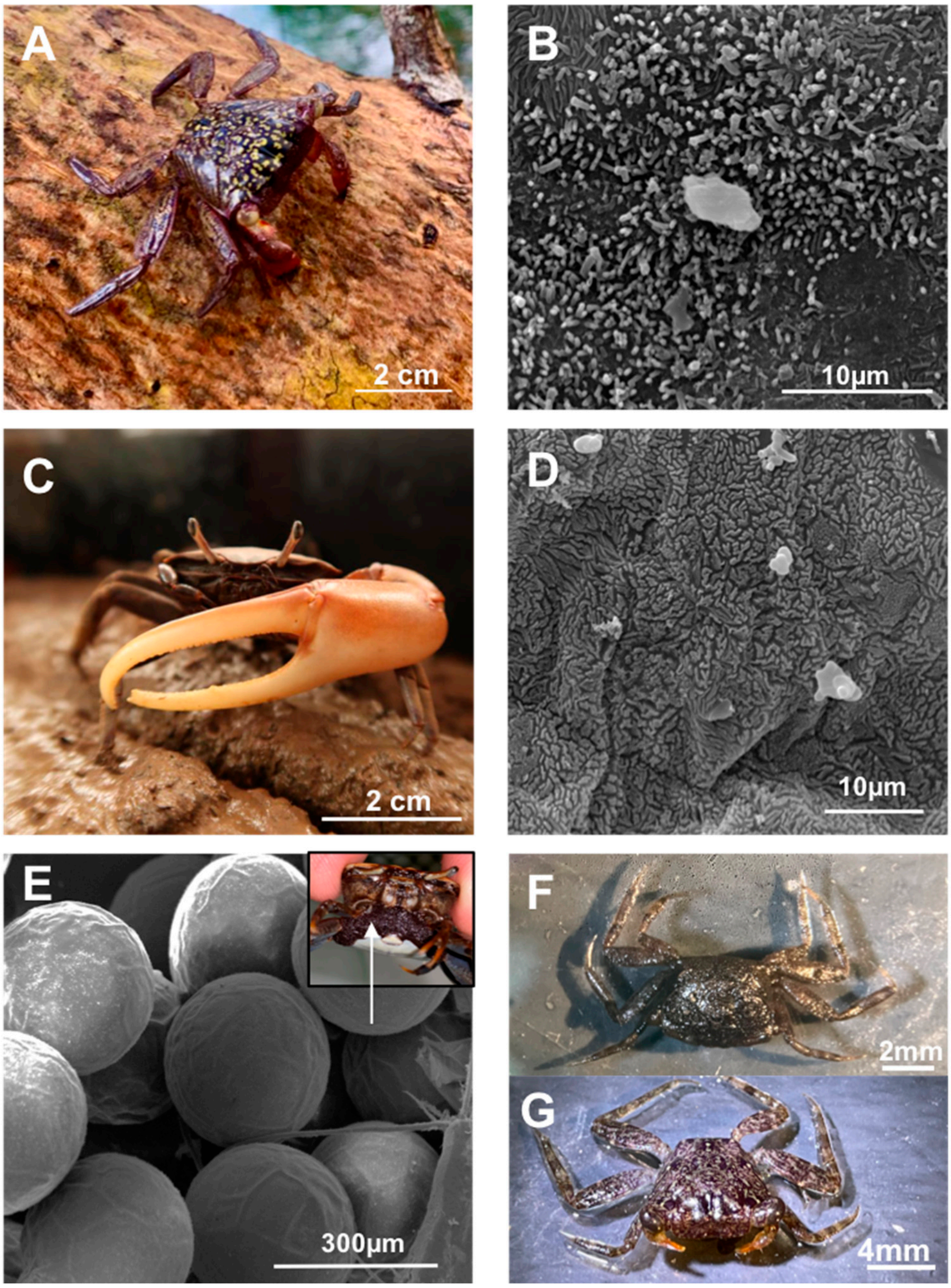

Supplementary data S1. Pictures of *A. pisonii* and *M. rapax*. Adult individual of *A. pisonii* (A) and *M. rapax* (C) have a bacterial biofilm covering their gill surface (B-D) as observed in this study for juveniles of both species. Ovigerous female of *A. pisonii* (insert) shows no bacteria on the surface of the eggs according to SEM. The juvenile of *M. rapax* (F) is entirely black, unlike the adult, while the juvenile of *A. pisonii* (G) already shows almost all the colors of the adult's carapace.

Supplementary data S2. Sequence of primer sets used.

	Sequences	PCR conditions used	References
8F<br>*1492R*	*5'-AGAGTTTGATCCTGGCTCAG*<br>*5'-GGTTACCTTGTTACGACTT*	*Denaturation at 94°C for 4 min, 30 cycles at 94°C for 1 min, at 52°C for 45s, and 72°C for 1.5 min, and elongation for 7 min at 72°C for 8F-1492R.*	*[34,35]*
CFB<br>*563-8F*	*5' -GGACCCTTTAAACCCAAT*	*Denaturation at 94°C for 4 min, 30cycles at 94°C for 1 min, at 52°C for 45s, and 72°C for 1.5 min, and elongation for 7 min at 72°C for 8F-CFB563.*	*[34,36]*
ROS<br>*537-8F*	*5'- CAACGCTAACCCCCTCC*	*Denaturation at 94°C for 4 min, 30 cycles at 94°C for 1 min, at 52°C for 45s, and 72°C for 1.5 min, and 7 min extension at 72°C for 8F-ROS537.*	*[34,38]*
1F<br>*5R*	*5'*<br>*TACCTGGTTGATCCTGCCAGTAG*<br>*5'* CTTGGCAAATGCTTTCGC	*Denaturation at 94°C for 4 min, 30 cycles at 94°C for 1 min, at 52°C for 45s, and 72°C for 1.5 min, and 7 min extension at 72°C for 1F-5R*	*[33*<br>*]*

Supplementary data S3. Taxonomic rank of adults and juveniles main ASVs for *A. pisonii* and *M. rapax*

Sequence of adults and juveniles (Aratus pisonii)	Most precise taxonomic rank	Sequence of adults and juveniles (Minuca rapax)	Most precise taxonomic rank
Ara-1/ARA-juv-1	Rhodobacteraceae	Min-1	Phyllobacteriaceae
Ara-2	Bacteroidetes	Min-2/ MIN-juv-4	Flavobacteriales
Ara-3/ARA-juv-5	Saprospiraceae	Min-3/ MIN-juv-5	Acidimicrobiales/Sva0996 marine group
Ara-4	Bacteroidetes	Min-4	Rhodobacteraceae
Ara-5	Flavobacteriales	Min-5	NS11-12
ARA-juv-2	Rhodobacteraceae	MIN-juv-1	Rhodobacteriaceae
ARA-juv-4	Chitinophagales	MIN-juv-2	Bacteroidia
ARA-juv-5	Saprospiraceae	MIN-juv-3	Flavobacteriales
ARA-juv-6	Flavobacteriales		
ARA-juv-7	Chitinophagales		

Yellow: Bacteroidia, blue: Alphaproteobacteria, red: Acidimicrobiia
